# The Effect of Maternal High-Fat or High-Carbohydrate Diet during Pregnancy and Lactation on Cytochrome P450 2D (CYP2D) in the Liver and Brain of Rat Offspring

**DOI:** 10.3390/ijms25147904

**Published:** 2024-07-19

**Authors:** Wojciech Kuban, Anna Haduch, Ewa Bromek, Agnieszka Basińska-Ziobroń, Kinga Gawlińska, Dawid Gawliński, Małgorzata Filip, Władysława A. Daniel

**Affiliations:** 1 Department of Pharmacokinetics and Drug Metabolism, Maj Institute of Pharmacology, Polish Academy of Sciences, 31-343 Kraków, Poland; kuban@if-pan.krakow.pl (W.K.); haduch@if-pan.krakow.pl (A.H.); bromek@if-pan.krakow.pl (E.B.); ziobron@if-pan.krakow.pl (A.B.-Z.); 2 Department of Drug Addiction Pharmacology, Maj Institute of Pharmacology, Polish Academy of Sciences, 31-343 Kraków, Poland; kinga.gawlinska@uj.edu.pl (K.G.); gawlin@if-pan.krakow.pl (D.G.); mal.fil@if-pan.krakow.pl (M.F.)

**Keywords:** cytochrome P450 2D (CYP2D), liver, brain, maternal diet, high-fat and high-carbohydrate diet, male offspring

## Abstract

Cytochrome P450 2D (CYP2D) is important in psychopharmacology as it is engaged in the metabolism of drugs, neurosteroids and neurotransmitters. An unbalanced maternal diet during pregnancy and lactation can cause neurodevelopmental abnormalities and increases the offspring’s predisposition to neuropsychiatric diseases. The aim of the present study was to evaluate the effect of maternal modified types of diet: a high-fat diet (HFD) and high-carbohydrate diet (HCD) during pregnancy and lactation on CYP2D in the liver and brain of male offspring at 28 (adolescent) or 63 postnatal days (young adult). The CYP2D activity and protein level were measured in the liver microsomes and the levels of mRNAs of *CYP2D1*, *2D2* and *2D4* were investigated both in the liver and brain. In the liver, both HFD and HCD increased the mRNA levels of all the three investigated *CYP2D* genes in adolescents, but an opposite effect was observed in young adults. The CYP2D protein level increased in adolescents but not in young adults. In contrast, young adults showed significantly decreased CYP2D activity. Similar effect of HFD on the *CYP2D* mRNAs was observed in the prefrontal cortex, while the effect of HCD was largely different than in the liver (the *CYP2D2* expression was not affected, the *CYP2D4* expression was decreased in young adults). In conclusion, modified maternal diets influence the expression of individual *CYP2D1*, *CYP2D2* and *CYP2D4* genes in the liver and brain of male offspring, which may affect the metabolism of CYP2D endogenous substrates and drugs and alter susceptibility to brain diseases and pharmacotherapy outcome.

## 1. Introduction

A mother’s diet during pregnancy and lactation is very important for the proper development of her child and has a long-term impact on its health. Fetal and neonatal exposure to unbalanced maternal nutrition can cause neurodevelopmental abnormalities and disrupt offspring physiology and behavior even in later life, resulting in increased susceptibility to many diseases, such as obesity, diabetes and neuropsychiatric disorders, including depression [1,2,3,4,5]. A cause of these disorders may involve epigenetic mechanisms, including DNA methylation, histone acetylation and microRNAs [6,7,8,9,10,11,12].

The CYP enzyme profile of the offspring is essentially determined by the genetic information passed down from the parents. The expression of CYP enzymes may be additionally altered by environmental factors, such as nutrition status, diets and drugs [13,14,15,16,17,18]. However, only a few studies have indicated that the mother’s diet can influence the expression and activity of offspring CYP enzymes through epigenetic mechanisms and fetal programming. It has been shown that consumption of a high-fat diet by female mice during pregnancy decreases the CYP3A protein level and activity in the livers of their 6-week-old offspring, which was directly related to an elevation in ERK phosphorylation and a declined expression of CAR nuclear receptor [19]. Another study indicated that a maternal high-fat diet during pregnancy changed the protein expression of CYP enzymes in the livers of newborn male mice. The expression of CYP3A and CYP2C11 decreased while the expression of CYP1A and CYP2E rose at 6 and 12 weeks of age. The level of total CYP2D protein did not change at these two time points [15], but individual CYP2D subfamily enzymes were not investigated. The amount of pregnane X receptor (PXR) translocated to the nucleus was reduced in the livers of infant male mice born to dams that consumed a high-fat diet. However, there was neither an increase in tumor necrosis factor-α or interleukin-1β nor a decrease in lithocholic acid. The authors suggest that the decreased expression of CYP3A and CYP2C11 enzymes is in close association with decreased expression of the nuclear receptor PXR in male offspring of mothers on a high-fat diet during pregnancy. 

Many different CYP2D enzymes have been found in numerous species, including humans and rodents, such as mice and rats. Humans have only one functional *CYP2D* gene, namely *CYP2D6*, and two pseudogenes (*CYP2D7* and *CYP2D8*), while in rodents there are a number of expressed *CYP2D* genes [20]. Mice have nine *Cyp2d* genes; therefore, measuring the total Cyp2d protein contents might hide alterations in a particular mouse Cyp2d protein [15,21]. Compared to mice, rats have six CYP2D enzymes (CYP2D1–5 and CYP2D18) exhibiting similar substrate/reaction specificity [22]. CYP2D1 and CYP2D2 are the most abundant forms in the rat liver, while CYP2D4 is primarily expressed in the rat brain [23,24]. The region- and cell-specific expression of brain CYP2D shows marked interspecies similarities with the highest CYP2D expression in the substantia nigra and cerebellum [25,26,27]. Thus, studying the expression and activity of individual rodent CYP2Ds is advisable to find out possible changes in their expression and activity produced by environmental factors, such as diets or drugs.

CYP2D enzymes are important in psychopharmacology since they may be engaged in the synthesis of the neurotransmitters dopamine and serotonin and in the metabolism of neurosteroids and neuroactive drugs [24,28]. The aim of the present study was to evaluate the effect of maternal-modified types of diet: a high-fat diet (HFD) and high-carbohydrate diet (HCD) during pregnancy and lactation on the expression of particular *CYP2D* genes (*CYP2D1*, *CYP2D2*, *CYP2D4*) in the brain (prefrontal cortex) and in the liver of male rat offspring at 28 or 63 postnatal days. The selected brain region has a significant role in cognitive processes, decision-making and emotional behavior and is involved in the pathology of neuropsychiatric disorders, such as depression, schizophrenia, autism, attention deficit hyperactivity disorder (ADHD) and drug dependence [29,30,31,32,33,34,35].

## 2. Results

### 2.1. The Influence of Maternal Diet on the Expression and Activity of CYP2D in the Liver of Adolescent and Adult Male Offspring

The effect of maternal modified types of diet: HFD and HCD ingested during pregnancy and lactation on the expression (the mRNA and protein level) of the two main liver CYP2D enzymes (CYP2D1, CYP2D2) and the main brain CYP2D enzyme (CYP2D4), and on the activity of CYP2D enzyme subfamily was measured in the liver of male offspring at 28 (adolescent) or 63 (young adult) postnatal days. The animals derived from mothers on the standard diet (STD) were used as controls.

The mRNA level of the analyzed *CYP2D1* and *CYP2D2* genes was higher in the liver than in the brain, as indicated by the number of cycles in the RT-PCR analysis (Ct ≈ 20 and 17.5 vs. Ct ≈ 32 and 31, respectively). Moreover, the mRNA level of *CYP2D1* and *CYP2D2* was higher (Ct ≈ 20 and 17.5, respectively) than that of *CYP2D4* (Ct ≈ 23) in the liver of the investigated male offspring (the average of Ct values for each experimental group are shown in Tables S1 and S2 of Supplementary Materials).

**Figure 1:**
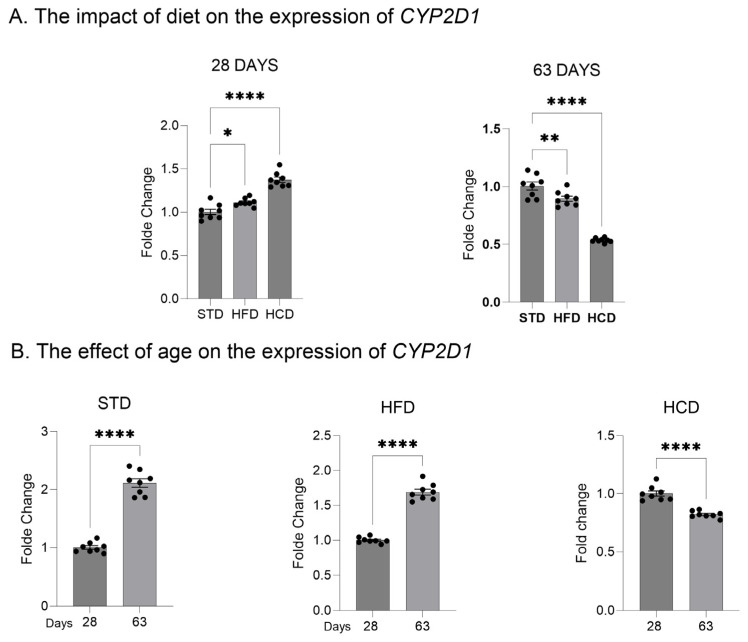
The effect of maternal high-fat diet (HFD) or high-carbohydrate diet (HCD) during pregnancy and lactation on the *CYP2D1* mRNA level in the liver of male rat offspring at postnatal days 28 and 63. The data are expressed as the mean ± S.E.M (n = 8). (**A**) The results were evaluated statistically using a one-way analysis of variance (ANOVA) followed by Dunnett’s multiple comparison test. (**B**) The results were analyzed using the unpaired Student’s *t*-test. Statistical significance is shown as * *p* < 0.05, ** *p* < 0.01; **** *p* < 0.0001. STD—male offspring from mothers on standard diet (control group); HFD—male offspring from mothers on high-fat diet; HCD—male offspring from mothers on high-carbohydrate diet.

**Figure 2:**
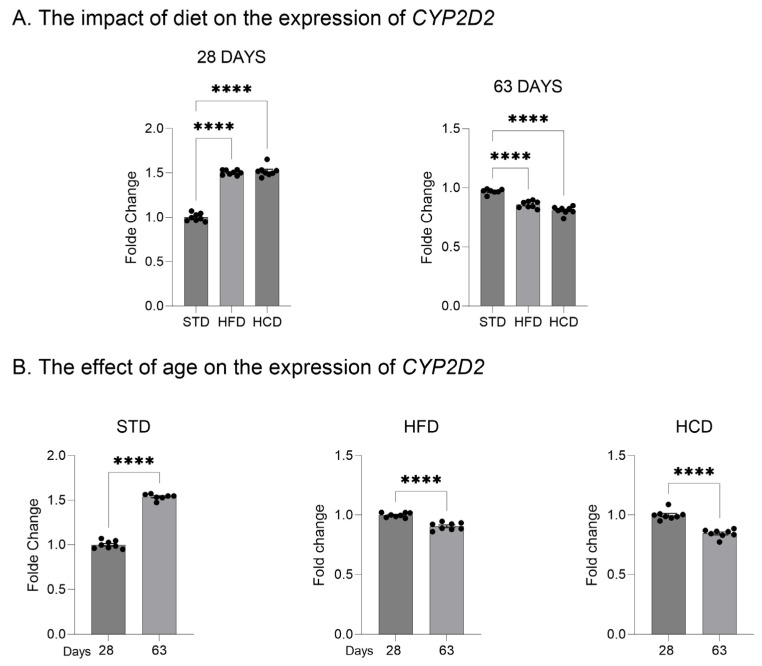
The effect of maternal high-fat diet (HFD) or high-carbohydrate diet (HCD) during pregnancy and lactation on the *CYP2D2* mRNA level in the liver of male rat offspring at postnatal days 28 and 63. The data are expressed as the mean ± S.E.M. (n = 8). (**A**) The results were evaluated statistically using a one-way analysis of variance (ANOVA) followed by Dunnett’s multiple comparison test. (**B**) The results were analyzed using the unpaired Student’s *t*-test. Statistical significance is shown as **** *p* < 0.0001. STD—male offspring from mothers on standard diet (control group); HFD—male offspring from mothers on high-fat diet; HCD—male offspring from mothers on high-carbohydrate diet.

**Figure 3:**
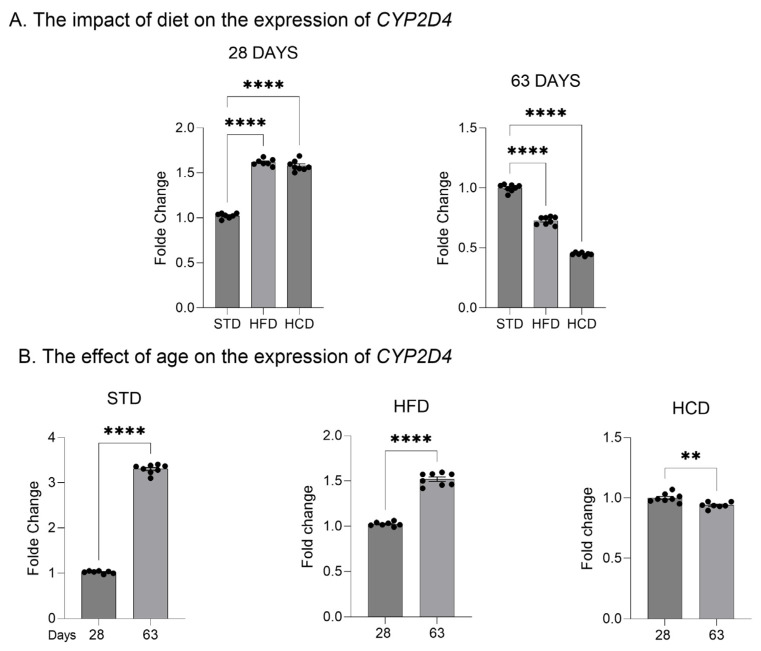
The effect of maternal high-fat diet (HFD) or high-carbohydrate diet (HCD) during pregnancy and lactation on the *CYP2D4* mRNA level in the liver of male rat offspring at postnatal days 28 and 63. The data are expressed as the mean ± S.E.M (n = 8). (**A**) The results were evaluated statistically using a one-way analysis of variance (ANOVA) followed by Dunnett’s multiple comparison test. (**B**) The results were analyzed using the unpaired Student’s *t*-test. Statistical significance is shown as ** *p* < 0.01; **** *p* < 0.0001. STD—male offspring from mothers on standard diet (control group); HFD—male offspring from mothers on high-fat diet; HCD—male offspring from mothers on high-carbohydrate diet.

In the livers of adolescent offspring, the modified maternal diets (both HFD and HCD) increased the mRNA levels of the three investigated *CYP2D* genes: *CYP2D1*, *CYP2D2* and *CYP2D4* (Figure 1A, Figure 2A and Figure 3A). In contrast, young adult offspring exposed to maternal HFD or HCD showed significantly decreased mRNA levels of *CYP2D1/2/4* genes in the liver (Figure 1A, Figure 2A and Figure 3A).

The *CYP2D1* mRNA level significantly increased with age (between 28 and 63 postnatal days) in the livers of male offspring descended from mothers on the standard and high-fat diets but declined in the offspring from mothers on HCD (Figure 1B). In the case of *CYP2D2* gene, a moderate increase in the *CYP2D2* mRNA between 28 and 63 postnatal days was observed only in the offspring from mothers on the standard diet, while a mild decrease was evoked by both modified diets (Figure 2B). Similarly to the *CYP2D1* gene, the hepatic *CYP2D4* mRNA significantly increased with age in male offspring from mothers on the standard and high-fat diets but slightly declined in the offspring from mothers on HCD (Figure 3B).

**Figure 4:**
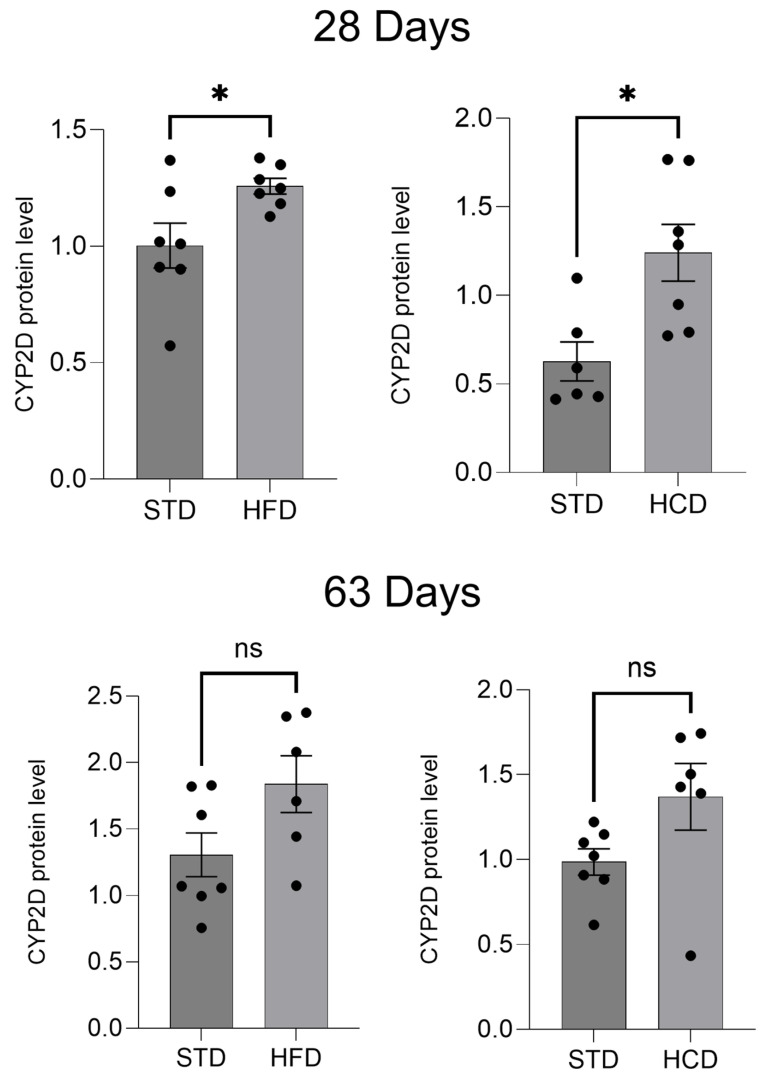
The effect of maternal high-fat diet (HFD) or high-carbohydrate diet (HCD) during pregnancy and lactation on the CYP2D protein level in the liver of male rat offspring at postnatal days 28 and 63. All values are the mean ± SEM of 6–7 animals. The results were evaluated statistically using the unpaired Student’s *t*-test of independent samples. Statistical significance is shown as * *p* < 0.05, ns—not significant. STD—male offspring from mothers on standard diet (control group); HFD—male offspring from mothers on high-fat diet; HCD—male offspring from mothers on high-carbohydrate diet. The original Western blot membranes are shown in Supplementary Materials (Figure S1).

The protein level of the investigated CYP2D subfamily was measured in the liver microsomes of the investigated offspring from mothers on STD, HFD or HCD. The Western blot analysis showed a significant increase in the CYP2D protein level in the livers at postnatal day 28 in adolescent offspring, made by the maternal-modified diets, both HFD and HCD (Figure 4, Figure S1 of Supplementary Materials). Such a tendency could be observed at postnatal day 63 in young adults.

**Figure 5:**
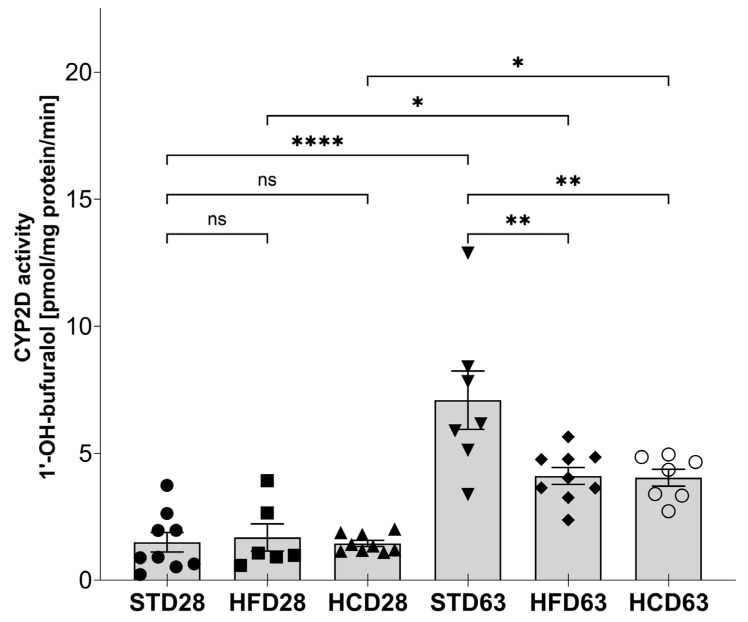
The effect of maternal high-fat diet (HFD) or high-carbohydrate diet (HCD) during pregnancy and lactation on the CYP2D activity in the liver of male rat offspring at postnatal days 28 and 63. The CYP2D activity was determined in liver microsomes using the CYP2D specific reaction, i.e., 1′-hydroxylation of bufuralol. The data are expressed as the mean ± S.E.M (n = 7–9). The results were evaluated statistically using a two-way analysis of variance (ANOVA) followed by Tukey’s multiple comparison test. Statistical significance is shown as * *p* < 0.05; ** *p* < 0.01; **** *p* < 0.0001, ns—not significant. STD28—male offspring from mothers on standard diet at postnatal day 28 (control group); HFD28—male offspring from mothers on high-fat diet at postnatal day 28; HCD28—male offspring from mothers on high-carbohydrate diet at postnatal day 28; STD63—male offspring from mothers on standard diet at postnatal day 63 (control group); HFD63—male offspring from mothers on high-fat diet at postnatal day 63; HCD63—male offspring from mothers on high-carbohydrate diet at postnatal day 63.

The CYP2D enzyme subfamily activity, measured in vitro as a rate of bufuralol 1′-hydroxylation in liver microsomes, was not altered by the modified maternal diets HFD or HCD in adolescents but was significantly reduced by those maternal diets in young adults (Figure 5). Moreover, the observed increase in the CYP2D activity between 28 and 63 postnatal days was greater in the offspring from mothers on STD than in the offspring from mothers on the modified diets HFD or HCD.

### 2.2. The Influence of Maternal Diet on the Expression of CYP2D Genes in the Brain of Adolescent and Adult Male Offspring

The effect of maternal diets (HFD or HCD) on the expression (mRNA) of the main brain CYP2D enzyme (CYP2D4), and comparatively of the two main liver CYP2D enzymes (CYP2D1 and CYP2D2) was measured in the brain of male offspring at 28 (adolescent) or 63 (young adult) postnatal days. The mRNA level of *CYP2D4* was higher (Ct ≈ 24) than that of *CYP2D1* (Ct ≈ 32) or *CYP2D2* (Ct ≈ 31) in the prefrontal cortex of the investigated male offspring, as indicated by the number of cycles in the RT-PCR analysis (Tables S1 and S2 of Supplementary Materials).

**Figure 6:**
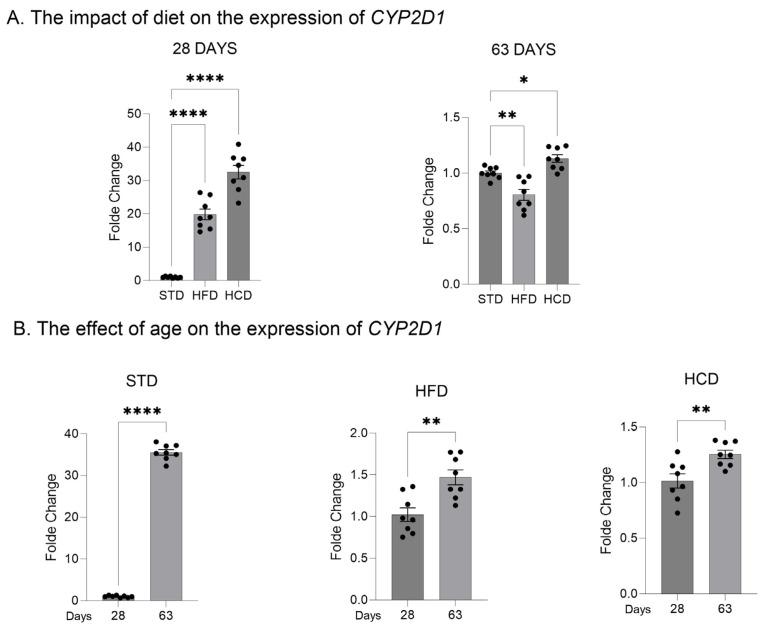
The effect of maternal high-fat diet (HFD) or high-carbohydrate diet (HCD) during pregnancy and lactation on the *CYP2D1* mRNA level in the prefrontal cortex of male rat offspring at postnatal days 28 and 63. The data are expressed as the mean ± S.E.M (n = 8). (**A**) The results were evaluated statistically using a one-way analysis of variance (ANOVA) followed by Dunnett’s multiple comparison test. (**B**) The results were analyzed using the unpaired Student’s *t*-test. Statistical significance is shown as * *p* < 0.05; ** *p* < 0.01; **** *p* < 0.0001. STD—male offspring from mothers on standard diet (control group); HFD—male offspring from mothers on high-fat diet; HCD—male offspring from mothers on high-carbohydrate diet.

**Figure 7:**
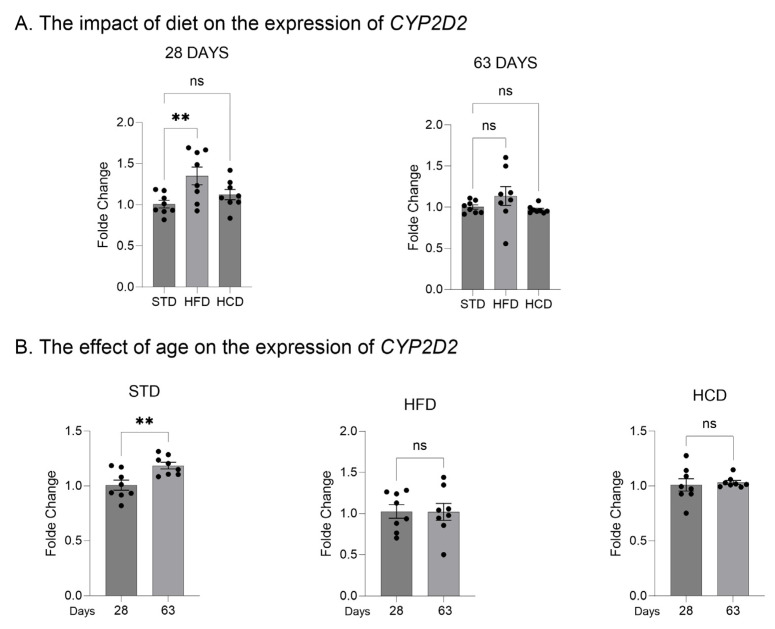
The effect of maternal high-fat diet (HFD) or high-carbohydrate diet (HCD) during pregnancy and lactation on the *CYP2D2* mRNA level in the prefrontal cortex of male rat offspring at postnatal days 28 and 63. The data are expressed as the mean ± S.E.M (n = 8). (**A**) The results were evaluated statistically using a one-way analysis of variance (ANOVA) followed by Dunnett’s multiple comparison test. (**B**) The results were analyzed using the unpaired Student’s *t*-test. Statistical significance is shown as ** *p* < 0.01, ns—not significant. STD—male offspring from mothers on standard diet (control group); HFD—male offspring from mothers on high-fat diet; HCD—male offspring from mothers on high-carbohydrate diet.

**Figure 8:**
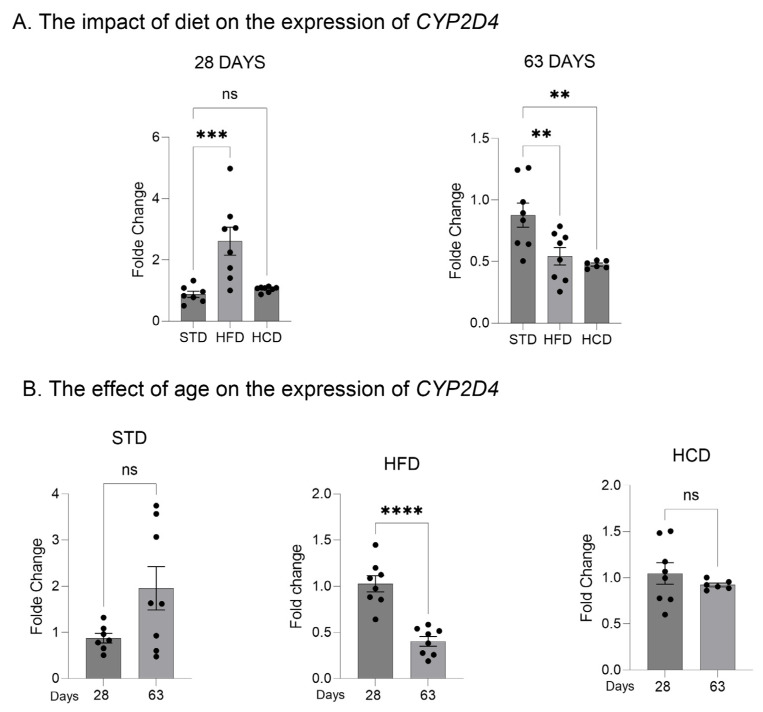
The effect of maternal high-fat diet (HFD) or high-carbohydrate diet (HCD) during pregnancy and lactation on the *CYP2D4* mRNA level in the prefrontal cortex of male rat offspring at postnatal days 28 and 63. The data are expressed as the mean ± S.E.M (n = 8). (**A**) The results were evaluated statistically using a one-way analysis of variance (ANOVA) followed by Dunnett’s multiple comparison test. (**B**) The results were analyzed using the unpaired Student’s *t*-test. Statistical significance is shown as ** *p* < 0.01; *** *p* < 0.001; **** *p* < 0.0001, ns—not significant. STD—male offspring from mothers on standard diet (control group); HFD—male offspring from mothers on high-fat diet; HCD—male offspring from mothers on high-carbohydrate diet.

In the prefrontal cortex, HFD increased the mRNA levels of all the three investigated *CYP2D* genes (*CYP2D1, CYP2D2* and *CYP2D4*) in adolescent offspring, as compared to adolescents from mothers on the standard diet (STD). In contrast, HFD decreased (*CYP2D1* and *CYP2D4*) or did not significantly affect (*CYP2D2*) those levels in young adult offspring (Figure 6A, Figure 7A and Figure 8A). For comparison, HCD increased only the *CYP2D1* mRNA in adolescent offspring (Figure 6A), not affecting other *CYP2D* mRNAs. The observed increase in the *CYP2D1* mRNA at 28 day was maintained at day 63 in young adults and was accompanied by the declined *CYP2D4* mRNA and unchanged *CYP2D2* mRNA level (Figure 7A and Figure 8A). 

The *CYP2D1* mRNA significantly increased with age (between 28 and 63 postnatal days) in male offspring from mothers on all the three types of diet, the rise being much higher in the offspring from mothers on the standard diet (Figure 6B). In the case of the *CYP2D2* gene, only a slight increase in its mRNA was noticed with age in the offspring from mothers on the standard diet (Figure 7B), whereas a moderate increase in the *CYP2D4* mRNA level was observed with age in the offspring from mothers on the standard diet (Figure 8B). In contrast, the *CYP2D4* mRNA level was reduced with age in the offspring from mothers on HFD, and no significant age-dependent change was seen in the offspring from mothers on HCD (Figure 8B).

Because of a very low weight of the prefrontal cortex in rats, the CYP2D protein level and enzymatic activity were not analyzed in this brain structure in the present experiment.

## 3. Discussion

This is the first report showing alterations in the expression of individual CYP2D enzymes in the liver and brain of male rat offspring, evoked by modified maternal diets. HFD and HCD during pregnancy and lactation affect the expression and activity of individual CYP2D enzymes in the liver and brain of male offspring, and the changes are organ-, CYP2D enzyme-, age- and diet type-dependent (Table 1).

As indicated by the number of cycles in the RT-PCR analysis, the mRNA level of the analyzed *CYP2D1* and *CYP2D2* genes was higher in the liver than in the brain of the investigated male offspring. Moreover, the mRNA level of liver *CYP2D1* and *CYP2D2* was higher than that of *CYP2D4*. Reverse relations between the *CYP2D1/2* and *CYP2D4* mRNA level were observed in the brain where the *CYP2D4* mRNA level was higher than that of *CYP2D1* or *CYP2D2*. These observations are consistent with those of Hiroi et al. [23] who could measure only the *CYP2D4* mRNA in the brain, while in the liver the mRNAs of all rat *CYP2D* genes were found. Thus, it can be assumed that in the liver the expression of *CYP2D1* and *CYP2D2* genes mainly translates into the CYP2D protein level and activity, while in the brain the expression of *CYP2D4* gene mainly results in the enzyme protein level and activity. Thus, the contribution of particular CYP2D enzymes in the alterations evoked by modified maternal diet may be different in the liver and brain.

In the liver, both HFD and HCD increased the mRNA levels of all the three investigated *CYP2D* genes, namely *CYP2D1*, *CYP2D2* and *CYP2D4*, in male offspring at postnatal day 28 (adolescent), but an opposite effect was observed in male offspring at postnatal day 63 (young adult). The *CYP2D* mRNA increases observed in adolescents were followed by enhanced CYP2D protein level but not CYP2D activity, which may suggest post-translational modification of the enzyme protein at postnatal day 28. On the other hand, the *CYP2D* mRNA decreases found in young adults from mothers on HFD or HCD were not accompanied by a corresponding reduction in the CYP2D protein level, which suggests protein stabilization evoked by both diets. Nevertheless, the enzyme activity declined, which implies post-translational enzyme modification produced by both diets at postnatal day 63. The above results differ from those of Tajima et al. [15] who did not observe any changes in total CYP2D protein level in male mice at 6 and 12 weeks of age whose mothers consumed a high-fat diet during pregnancy, which may result from interspecies differences in enzyme regulation, postnatal day, and specific diet composition.

The mechanism of the observed diet-evoked changes in the CYP2D enzyme expression and activity in the liver has not been investigated as yet; however, abnormal diets are known to induce epigenetic mechanisms (such as DNA methylation, histone modification, and miRNA effect), which affect gene expression or protein stability and lead to metabolic diseases including hepatocellular carcinoma [12,36]. Since liver CYP2D enzymes are important for drug metabolism, interindividual differences in their expression level and functioning produced by nutrition-induced epigenetic changes may lead to diversified drug response, in particular antidepressants, antipsychotics, and cardiovascular drugs [37]. 

Our previous studies showed that maternal HFD or HCD induced molecular alterations also in the brain of male offspring, leading to behavioral emotional symptoms representative of depression and substance-use disorders and disturbances in cognitive processes in animal models [2,36]. Recent research has indicated that maternal HFD influences the expression of neuronal markers specific to excitatory and inhibitory cortical neurons and myelin-related genes in the brain prefrontal cortex of rat offspring [1,5], which may contribute to the observed diet-produced behavioral syndromes. 

Since CYP2D enzymes are also present in the brain, where they can synthesize neurotransmitters (dopamine from tyramine and serotonin from 5-methoxytryptamine) and catalyze hydroxylation of neurosteroids [28], we found it interesting to test whether changes in CYP2Ds observed in the liver occur also in the brain. To this aim, we chose the prefrontal cortex as a brain structure significant for cognitive function and decision-making and involved in the pathogenesis of the above-mentioned mental disorders. We found that maternal HFD produced similar changes in the mRNAs of the investigated *CYP2D* genes as in the liver, i.e., an increase at postnatal day 28 and a decrease at postnatal day 63 (Table 1). The exception was *CYP2D2* gene (far less expressed in the brain than in the liver), the expression of which was not changed in young adults. 

Compared to HFD, the effect of HCD on brain CYP2Ds was largely different than on liver CYP2Ds. The expression of CYP2D2 gene was not affected by HCD, neither in adolescent nor in young adult offspring, while the expression of *CYP2D4* gene (the main CYP2D in the brain) was decreased in adult offspring only. As the regulation of expression of brain *CYP* genes may differ from that in the liver [38,39,40,41,42,43,44,45], it remains to be examined whether the brain CYP2D protein level and activity undergo similar alterations as those observed in the liver at the same postnatal day. Answering this question seems of importance, since abnormal functioning of the alternative pathways of neurotransmitter synthesis (dopamine, serotonin) or neurosteroid metabolism catalyzed by CYP2D could affect the predisposition of offspring to mental disorders [28]. It is conceivable that diet-induced epigenetic mechanisms evoke molecular and morphological alterations in the brain, which modify the development of neurotransmitter systems essential for optimal function of the central nervous systems. Therefore, a well-balanced diet is recommended, especially during pregnancy and lactation, to provide optimal conditions for brain development and healthy offspring and to avoid increased susceptibility to neuropsychiatric disorders. 

In addition, it should be emphasized that the effect of the offspring maturation between postnatal day 28 and 63 on the CYP2Ds’ expression in the liver and brain depended on the type of maternal diet (STD, HFD, HCD) and particular CYP2D enzyme. The expression of CYP2D enzymes is known to increase with age in humans and rats at physiological conditions [27,46,47]. However, animals from mothers on the modified diets showed less elevated expression of *CYP2D* genes or unchanged expression or even decreased *CYP2D* genes’ expression in the period between adolescence and adulthood, compared to the offspring from mothers on the standard diet. Thus, the modified maternal diets hamper the proper development of CYP2D enzymatic function to effectively deal with the biotransformation of foreign substances (drugs) in the liver and metabolism of endogenous substrates in the brain, such as neurosteroids and neurotransmitter precursors. Therefore, it seems possible that the above-described biochemical alterations produced by maternal-modified diets in cytochrome P450 may contribute to the observed increased predisposition to brain disorders among offspring from mothers on the high-fat or high-carbohydrate diets.

The reaction of bufuralol 1′-hydroxylation, which is widely used as a marker reaction for CYP2D subfamily enzymes in humans and rodents [48,49], was applied in our study for measuring the CYP2D activity in rat liver. Although other CYP enzymes can catalyze this reaction [50,51,52], kinetic studies and experiments carried out on liver microsomes using specific CYP2D inhibitors or anti-CYP2D antibodies clearly indicated the main, highly specific contribution of CYP2D enzymes to the catalysis of bufuralol 1′-hydroxylation [22,46,50,52]. Another limitation of this study is performing investigations only on male rats and in one brain structure. Further studies are scheduled to find out whether/how a modified maternal diet affects cytochrome P450 in female offspring. Moreover, it is advisable to investigate other brain regions involved in mental disorders, in respect to biochemical and functional alterations in the CYP2D subfamily enzymes, to find possible engagement of these enzymes in the diet-induced abnormalities in emotional processes. 

## 4. Materials and Methods

### 4.1. Animals

Wistar rats (Charles River, Sulzfeld, Germany) were housed in standard plastic cages in an animal colony room maintained at 22 ± 2 °C and 55 ± 10% humidity under a 12 h light/dark cycle (lights on at 6.00 a.m.). Animals had free access to food and water. Nulliparous female rats (200–240 g), after the acclimatization period and during the proestrus phase, were mated with males. The gestation was confirmed by examining vaginal smears for the presence of sperm. Dams were individually housed and randomly assigned to two groups: standard diet (STD; 13% fat, 3.4 kcal/g; VRF1; Special Diets Services, Witham, UK), high-fat diet (HFD; 60% fat, 5.31 kcal/g; C1057 mod.; Altromin, Lage, Germany) or high-carbohydrate diet (HCD; 70% carbohydrate: rich in 40% sucrose, 12% fat, 18% protein, 3.77 kcal/g; C1010). Animals had free access to the diets during pregnancy (21 days) and lactation (21 days) [2]. After weaning, offspring at postnatal day 22 were separated according to sex, housed 5 per cage, and switched to an STD. Male offspring at 28 (adolescent) or 63 postnatal days (young adult) were used in this study (n = 7–9 rats/group). The present study was carried out in accordance with the European Union Directive 2010/63/EU and with the approval from the Local Ethics Commission at the Maj Institute of Pharmacology, Polish Academy of Sciences, Kraków, Poland (the permission number: 1270/2015, 17 December 2015 and 18/2020, 23 January 2020). 

### 4.2. Chemicals

Reagents for determining the activity of CYP2D enzymes in liver microsomes (glucose-6-phosphate, glucose-6-phosphate-dehydrogenase, NADP) and specific CYP2D substrate and its metabolite (bufuralol and 1′-hydroxybufuralol) came from Sigma (St. Louis, MO, USA). SignalBoostTMImmunoreaction Enhancer Kit used for dilution of primary and secondary antibodies was supplied by Millipore (Burlington, MA, USA). Laemmli sample buffer used for dilution of samples was obtained from Bio-Rad (Hercules, CA, USA). RNA was isolated with Total RNA Mini kit from A&A Biotechnology (Gdynia, Poland). Life Technologies (Carlsbad, CA, USA) provided a High-Capacity cDNA Reverse Transcription Kit, TaqMan assay and the TaqMan Gene Expression Master Mix. Merck (Darmstadt, Germany) provided the organic solvents with HPLC purity.

### 4.3. Tissue Preparation and Determination of CYP2D Activity 

Livers and brains were dissected; the brain prefrontal cortex was separated in accordance with the rat brain atlas, frozen in dry ice and stored at −80 °C [53]. Liver microsomal fraction was prepared by differential centrifugation using standard conditions involving homogenization in a 20 mM Tris/KCl buffer at pH 7.4, followed by washing with 0.15 M KCl. The CYP2D activity was determined in liver microsomes using the CYP2D specific reaction, i.e., 1′-hydroxylation of bufuralol at a linear dependence of product formation on time, substrate and protein concentration [54]. The reaction proceeded in a system containing liver microsomes (0.5 mg of protein/mL), potassium phosphate buffer (2 mM, pH = 7.4), and NADPH generating system (1.6 mM NADP, 4 mM MgCl_2_ 5 mM glucose 6-phosphate and 2.5 U glucose 6-phosphate-dehydrogenase) in every sample. Bufuralol was added to the incubation medium containing liver microsomes at a concentration of 10 µM to the final volume of 0.4 mL. The amount of 1′-hydroxybufuralol formed from bufuralol was measured by an HPLC method with fluorometric detection.

### 4.4. Evaluation of CYP2D Protein in Liver Microsomes 

Because of a very low weight of the prefrontal cortex, the CYP2D protein level and enzymatic activity were not analyzed in this brain structure. The CYP2D protein levels in liver microsomes were quantified by Western blotting, as previously described [54]. Microsomal proteins (10 μg) were separated using an SDS polyacrylamide gel electrophoresis, and then the protein bands were transferred onto nitrocellulose membranes (Amersham Protran, Merck KGaA, Darmstadt, Germany). The polyclonal rabbit anti-human CYP2D6 antibody (Fine Test, Wuhan, China) was used as a primary antibody for CYP2D enzymes in liver microsomes. Horseradish peroxidase-labeled goat anti-rabbit IgG was used as a secondary antibody (Vector Laboratories, Burlingame, CA, USA). For the estimation of Hsp90 level, the primary rabbit polyclonal anti-rat Hsp90 antibody (Santa Cruz, CA, USA) and goat anti-rabbit antibody (Vector Laboratories, Burlingame, CA, USA) were used. Human cDNA-expressed recombinant protein CYP2D6 ~1 µg (Gentest Corp. Woburn, MA, USA) was used as the standard. The band intensity of CYP2D protein was evaluated with the Luminescent Image Analyzer LAS-1000 and Image Gauge 3.11 programs (Fuji Film, Tokyo, Japan). The collected data were normalized to protein loading based on the Hsp90 levels.

### 4.5. Examination of the Expression of Gene Coding for CYP2D Enzymes in the Liver and Brain

Total RNA was extracted from each sample using approximately 50 mg of frozen liver or selected brain structures with the Total RNA Mini Plus kit (A&A Biotechnology, Gdynia, Poland). For reverse transcription, about 2 µg of total RNA from each tissue sample was converted to cDNA according to the manufacturer’s protocol (High-Capacity cDNA Reverse Transcriptase kit, Applied Biosystems, Waltham, MA, USA). The resulting cDNA served as the template for quantitative real-time PCR, using the TaqMan Gene Expression Assay with TaqMan Fast Advanced Master Mix, TaqMan probes [*CYP2D1* (Rn01775090_mH), *CYP2D2* (Rn00562419_m1), *CYP2D4* (Rn00593393_m1)]. Specific probes for the β-actin gene (Actb; Rn00667869) were used as an internal reference for assessing *CYP* expression, all sourced from Applied Biosystems (Waltham, MA, USA). Amplification was performed on the QuantStudio 12K Flex real-time PCR system (Thermo Fisher Scientific Inc., Waltham, MA, USA), following 40 cycles of standard conditions suitable for TaqMan-based assays in relative quantification mode. The QuantStudio 12K Flex system software (version 1.4) calculated the threshold cycle (Ct) values for the *CYP* and *Actb* genes. The comparative ΔCt method was used to evaluate the expression of individual *CYP* genes relative to *Actb* expression. Relative-fold mRNA content was determined using the 2^−ΔΔCt^ formula, according to Livak and Schmittgen [55,56].

### 4.6. Data Analysis

The statistical significance of alterations in CYP2D enzyme activity, mRNA levels of CYP2D genes, and CYP protein levels in rat liver and brain samples was evaluated using GraphPad Prism 10 software (GraphPad Software, Inc., La Jolla, CA, USA). The Shapiro–Wilk test was employed to assess normality. All sample groups had normally distributed results. The results were analyzed statistically using a one-way ANOVA followed by Dunnett’s multiple comparison test (the effect of diet on the individual *CYP2D* mRNAs), a two-way ANOVA followed by Tukey’s multiple comparison test (the effect of diet and age on the total CYP2D activity) or the unpaired Student’s *t*-test to evaluate the effect of diet on the total CYP2D protein level and the effect of age on the individual *CYP2D* mRNAs, as described in figure legends. Results are presented as the mean ± SEM, and were considered significant at *p* ≤ 0.05.

## 5. Conclusions

The modified maternal diets, HFD and HCD, fed during pregnancy and lactation affected the expression of individual *CYP2D* genes (*CYP2D1*, *CYPD2*, *CYPD4*) in the liver and brain of male rats, which may lead to a decrease in the rate of metabolism of drugs, which are CYP2D substrates in the liver of young adults. A similar effect may occur in the brain where CYP2D-mediated local drug biotransformation and metabolism of endogenous neuroactive substrates take place. The biosynthesis of monoaminergic neurotransmitters and hydroxylation of neurosteroids may slow down in young adults, which requires additional biochemical and functional investigation. Further molecular studies are necessary to find the mechanisms (epigenetic) responsible for the observed alterations in CYP2D enzymes’ expression and activity in the liver and brain. It cannot be excluded that the alterations produced in the cytochrome P450 by maternal-modified diets increase the susceptibility of offspring to brain disorders and impact pharmacotherapy outcome. It is also advisable to find out whether/how a modified maternal diet affects cytochrome P450 in female offspring.

## Figures and Tables

**Table 1:** The effect of maternal high-fat diet (HFD) or high-carbohydrate diet (HCD) during pregnancy and lactation on cytochrome P450 2D (CYP2D) enzymes in the liver and prefrontal cortex of male rat offspring (based on the results presented in Figure 1, Figure 2, Figure 3, Figure 4, Figure 5, Figure 6, Figure 7 and Figure 8).

Diets/Postnatal Day	Maternal High Fat Diet (HFD) vs. Control Standard Diet (STD)						Maternal High-Carbohydrate Diet (HCD) vs. Control Standard Diet (STD)					
	28-Day Offspring			63-Day Offspring			28-Day Offspring			63-Day Offspring		
*CYP2Ds*	2D1	2D2	2D4	2D1	2D2	2D4	2D1	2D2	2D4	2D1	2D2	2D4
Prefrontal cortex mRNA	↑	↑	↑	↓	n.s.	↓	↑	n.s.	n.s.	↑	n.s.	↓
Liver mRNA	↑	↑	↑	↓	↓	↓	↑	↑	↑	↓	↓	↓
Liver protein	↑			n.s.			↑			n.s.		
Liver activity	n.s.			↓			n.s.			↓		

↑ increase, ↓ decrease, n.s. not significant.

## Data Availability

Data are contained within the article.

## Supplementary Materials

The following supporting information can be downloaded at: https://www.mdpi.com/article/10.3390/ijms25147904/s1.
