# Supplementary material for: The Effect of Maternal High-Fat or High-Carbohydrate Diet during Pregnancy and Lactation on Cytochrome P450 2D (CYP2D) in the Liver and Brain of Rat Offspring
Source: Int J Mol Sci. 2024 Jul 19;25(14):7904. doi: 10.3390/ijms25147904 (PMC11276948; doi:10.3390/ijms25147904)
Supplement: Supplementary file 1 [file ijms-25-07904-s001.zip › ijms-3073101-supplementary.pdf]

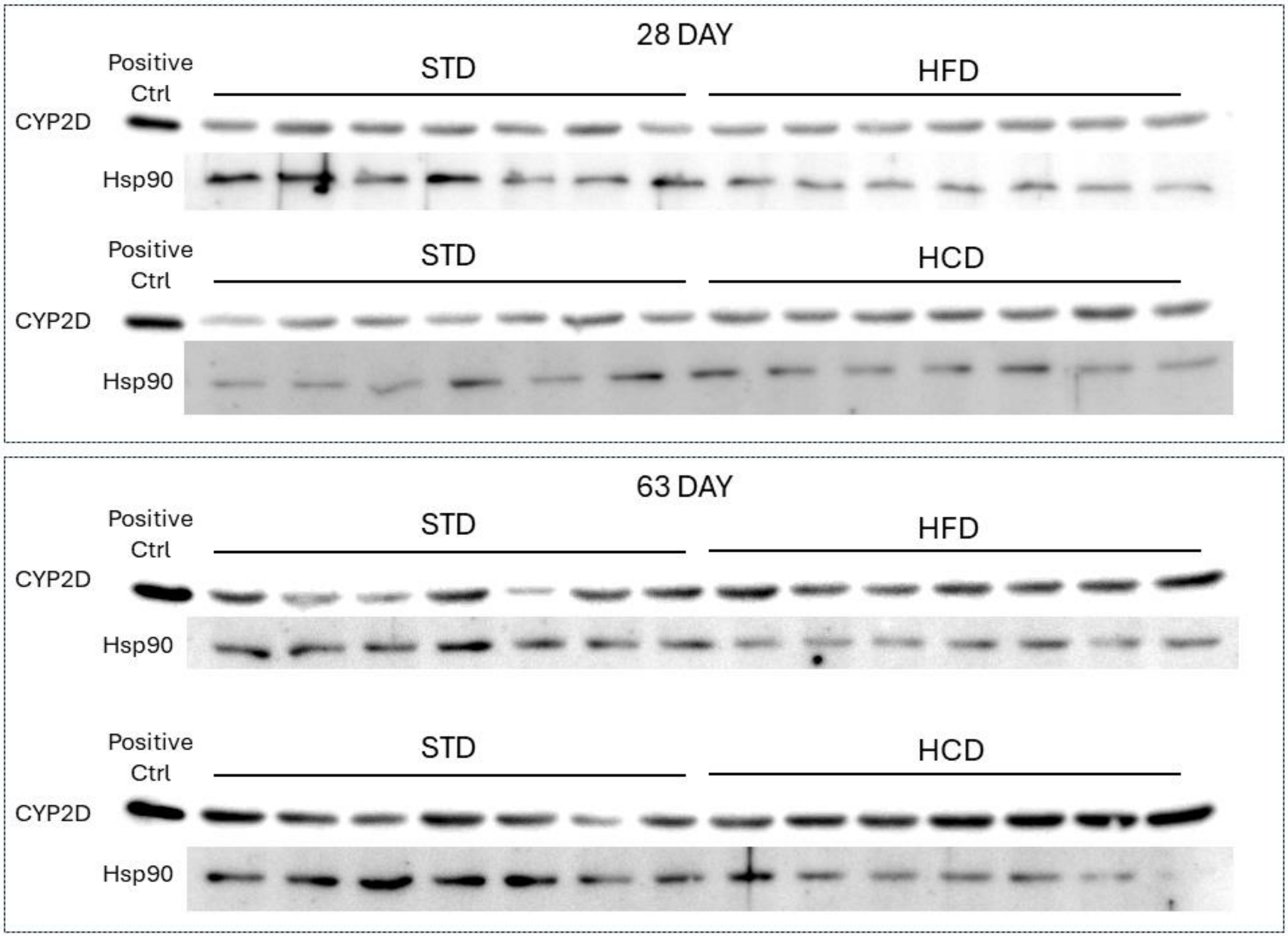

Figure S1. The effect of maternal high-fat diet (HFD) or high-carbohydrate diet (HCD) during pregnancy and lactation on cytochrome CYP2D protein level in the liver of male rat offspring at postnatal day 28 and 63 (original membranes of Western blotting). STD – standard diet, Positive Ctrl – Positive control.

Table S1. The number of cycles in the RT-PCR analysis of CYP2D mRNAs in the liver of male rat offspring at postnatal day 28 and 63. Shown as the mean (n = 8) of Ct values for liver cDNA samples.

Diets	CYP2D1		CYP2D2		CYP2D4	
	28 Day	63 Day	28 Day	63 Day	28 Day	63 Day
STD	19,703	19,760	17,181	17,645	23,170	22,577
HFD	20,093	19,488	17,135	17,430	23,055	22,603
HCD	19,640	19,858	16,971	17,147	22,906	22,843

Table S2. The number of cycles in the RT-PCR analysis of CYP2D mRNAs in the brain prefrontal cortex of male rat offspring at postnatal day 28 and 63. Shown as the mean (n = 8) of Ct values for cDNA samples of the prefrontal cortex.

Diets	CYP2D1		CYP2D2		CYP2D4	
	28 Day	63 Day	28 Day	63 Day	28 Day	63 Day
STD	34,661	29,746	31,078	31,071	24,857	24,970
HFD	30,976	30,219	31,273	31,086	24,533	24,808
HCD	29,903	29,620	31,165	31,162	24,275	24,579
